# Delayed effects of acute whole body lethal radiation exposure in mice pre-treated with BBT-059

**DOI:** 10.1038/s41598-020-63818-7

**Published:** 2020-04-22

**Authors:** Neel K. Sharma, Gregory P. Holmes-Hampton, Vidya P. Kumar, Shukla Biswas, Kefale Wuddie, Sasha Stone, Zemenu Aschenake, William L. Wilkins, Christine M. Fam, George N. Cox, Sanchita P. Ghosh

**Affiliations:** 1 Armed Forces Radiobiology Research Institute, Uniformed Services University of the Health Sciences, Bethesda, MD 20889 USA; 2 Bolder Biotechnology, Boulder, CO 80301 USA

**Keywords:** Ageing, Predictive markers

## Abstract

The threat of nuclear exposure is heightened and it is imperative to identify potential countermeasures for acute radiation syndrome. Currently no countermeasures have been approved for prophylactic administration. Effective countermeasures should function to increase survival in the short term as well as to increase the overall prognosis of an exposed individual long term. Here we describe the use of a promising radiation countermeasure, BBT-059, and the results of a long term mouse study (up to 12 months) in the male CD2F1 strain using ^60^Co gamma irradiation (~0.6 Gy/min, 7.5–12.5 Gy). We report the dose reduction factor of 1.28 for BBT-059 (0.3 mg/kg) compared to control administered 24 h prior to irradiation. In the long term study animals showed accelerated recovery in peripheral blood cell counts, bone marrow colony forming units, sternal cellularity and megakaryocyte numbers in drug treated mice compared to formulation buffer. In addition, increased senescence was observed in the kidneys of animals administered control or drug and exposed to the highest doses of radiation. Decreased levels of E-cadherin, LaminB1 and increased levels of Cyc-D and p21 in spleen lysates were observed in animals administered control. Taken together the results indicate a high level of protection following BBT-059 administration in mice exposed to lethal and supralethal doses of total body gamma-radiation.

## Introduction

In today’s geopolitical climate the threat of nuclear exposure either through intentional or accidental means is heightened. To this end the identification and development of effective radiation countermeasures is critical. Currently 3 drugs have United States Food and Drug Administration (FDA) approval for treating radiation exposure, Neupogen (Filgrastim), Neulasta (pegfilgrastim) and Leukine (sargramostim)^1–3^. All of these drugs have been approved for the mitigation of symptoms related to acute radiation syndrome (ARS) but no compounds have been approved as prophylactic countermeasures. For emergency first responders or war fighters tasked with a mission that includes entering a contaminated field prophylactic countermeasures could provide vital protection in cases where exposure is necessary to ensure public safety or to accomplish a mission. In order to effectively study potential countermeasures in animal models, one should consider both the efficacy of the compound in increasing survival at the primary end point of exposure as well as any delayed effects of acute radiation exposure (DEARE) that might arise. Because it is not feasible to test potential radiation countermeasures in humans, it is necessary to conduct these experiments in animal models using the guidance of the FDA animal rule^4^ which acts as a surrogate for the human response.

Recently we identified a promising radiation countermeasure BBT-059 (Bolder Biotechnology, Boulder, CO), a pegylated construct of the 20 kDa protein Interleukin 11 (IL-11). IL-11 was isolated in 1990^5^ and was demonstrated to act in anti-inflammatory processes as well as possessing hematopoietic proliferative properties^6^. Recombinant human IL-11 (rhIL-11) was found to be well tolerated in animal test systems but severe adverse effects have been noted in humans^6^ with daily dosing. To this end the long acting, pegylated IL-11 was developed by Bolder Biotechnology^7,8^ and was shown to remain bioactive up to 10 days in rats with a single dose. The compound was synthesized via site specific pegylation at a C-terminus cysteine residue which was added to the IL-11 protein (PEG-*179 C)^8^.

In a recent publication, we conducted experiments demonstrating enhanced survival in male CD2F1 mice across a wide range of times of administration (24 h prior to TBI up to 24 h after TBI) in reference to total body irradiation (TBI) exposure^9^. In this report we identified an optimal dose of administration for the compound (0.3 mg/kg) and demonstrated an accelerated recovery in bone marrow colony forming units, peripheral blood cell counts, sternal cellularity, megakaryocyte numbers, and circulating levels of serum biomarkers. These studies monitored the status of the animals and indicated parameters for 30 days following TBI.

Survivors of ARS, both those administered a countermeasure and those who are not, may experience delayed effects of acute radiation exposure (DEARE), DEARE can manifest chronic illnesses that affect multiple organ systems^10^. There is a need to better understand the underlying mechanisms that result in long-term syndromes that occur in multiple organs, as well to identify novel therapies that both improve the initial insult of TBI as well as the lasting DEARE.

DEARE was studied in an early publication by Lawrence and Tennant^11^ and since that time a number of studies to further our understanding have been published^10,12–20^. Several of these studies have addressed DEARE in irradiated animals without the use of radiation countermeasures at set time points relative to TBI. These studies can offer valuable insights into changes that occur as a result of exposure to ionizing radiation including fibrosis in major organs^10^ and significant loss of long term repopulating potential in bone marrow^17^.

Radiation is one of the stress factors that induces cellular senescence. Many studies have demonstrated that radiation induced cellular senescence often progresses to organ disease. Studies have demonstrated that a local radiation dose of 20 and 17.5 Gy induced cellular senescence in the heart and lung, respectively^21,22^. Aratani *et al*. (2018) reported cellular senescence in irradiated kidney with increased SA-β-gal activity and absence of Ki-67, a DNA proliferation marker^23^. Lamin B1, a protein from the nuclear lamina, has been reported to decrease in cells undergoing senescence^24^. CycD, a cell cycle protein, P21, cyclin dependent kinase inhibitor 1, and P16, cyclin dependent kinase inhibitor 2 A have all been implicated in cellular senescence because of their involvement in cell cycle progression^25^. Reduced recruitment to the cell surface following exposure to ionizing radiation has been reported for E-cadherin, a transmembrane protein,^26^. E-cadherin is found in epithelial tissue and its expression is associated with the epithelial-mesenchymal transition^27^. In the current study, animals are being kept for long-term analysis and accordingly senescence is an important process.

The effectiveness of a radiation countermeasure can be determined by dose reduction factor (DRF) which is a method for quantifying the efficiency of a radiation countermeasure. In these studies, the dose of radiation resulting in 50% survival for animals administered a countermeasure and the dose resulting in 50% survival for its vehicle are determined and the ratio of these value is used to quantify the effectiveness of the countermeasure^28^. In the current study we report the DRF for BBT-059 and the long term (up to 12 months) health effects for the surviving animals administered BBT-059 or its vehicle pre-exposure to various lethal and supralethal doses of whole body gamma-radiation.

## Results

### Dose reduction factor of BBT-059

**Figure 1:**
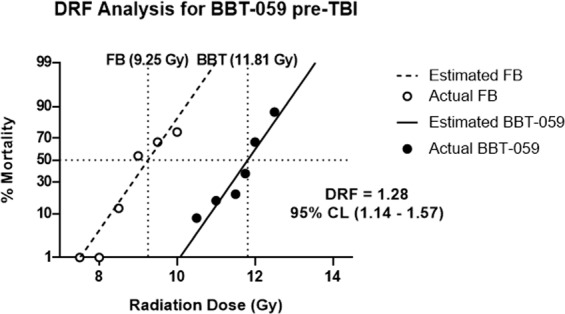
Dose reduction factor (DRF) determination. Mice were administered either FB or BBT-059 (0.3 mg/kg) 24 h prior to TBI at various doses. Radiation doses for the FB group were 7.5, 8.0, 8.5, 9.0, 9.5, and 10 Gy and radiation doses for the BBT-059 were 10.5, 11.0, 11.5, 11.75, 12.0, and 12.5 Gy. Probit analysis was conducted for each group and LD50/30 values of 9.25 and 11.81 Gy were determined for the groups administered FB and BBT-059 respectively. This ratio, BBT-059/FB gives a DRF value of 1.28 with a 95% confidence interval of 1.14–1.57. n = 24 mice per group.

At the conclusion of the 30 day study, survival of the animals was as noted in Table 1. Probit statistical analysis (Fig. 1) demonstrated an LD50/30 for the animals administered formulation buffer (FB) of 9.25 Gy and an LD50/30 of the animals administered BBT-059 of 11.81 Gy. Taking the ratio of LD50/30 of BBT-059: LD 50/30 of FB, DRF was found to be 1.28 (95% confidence interval of 1.14–1.57).

**Table 1:** Survival of animals in the study.

Group #	Survival of animals				
	Radiation Dose	Drug/Formulation Buffer	# Surviving (30 days)	# Surviving (6 months)	# Surviving (12 months)
naïve	0 Gy	—	15/15	10/10	4/5
1	7.5 Gy	Formulation Buffer	24/24	N/A	N/A
2	8.0 Gy	Formulation Buffer	24/24	N/A	N/A
3	8.5 Gy	Formulation Buffer	21/24	N/A	N/A
4	9.0 Gy	Formulation Buffer	11/24	N/A	N/A
5	**9.5 Gy**	**Formulation Buffer**	8/24	8/10	4/4
6	**10.0 Gy**	**Formulation Buffer**	6/24		
7	**10.5 Gy**	**BBT-059**	22/24	9/10	4/4
8	11.0 Gy	BBT-059	20/24	N/A	N/A
9	**11.5 Gy**	**BBT-059**	19/24	10/10	3/5
10	11.75 Gy	BBT-059	15/24	N/A	N/A
11	**12.0 Gy**	**BBT-059**	8/24	9/10	1/4
12	12.5 Gy	BBT-059	3/24	*N/A	N/A

For the initial 30 day DRF study survival of all groups is shown. Animals in **bold** were kept up to 12 months for the long term study. Animals from the 2 highest doses of radiation that were administered FB prior to TBI were pooled into a common group. Animals from the group administered BBT-059 prior to TBI at the highest radiation dose (12.5 Gy) were used for the 1 month collection, no animals from the 12 Gy dose of radiation were used at this time point but the survivors were studied at 6 at 12 months.

### Long term survival for DEARE studies

For the DEARE (Table 1) studies, animals from the two highest doses of radiation (9.5 and 10.0 Gy) that were administered FB were grouped into a single group, referred to as the FB group; in addition, three distinct groups of animals administered BBT-059 24 h prior to TBI (n = 10 for each group) were kept including those irradiated at 10.5 Gy, 11.5 Gy, and 12 Gy for long term studies (6 months and 12 months collection points), these groups will be referred to as 10.5 Gy BBT-059, 11.5 Gy BBT-059, and 12.0 Gy BBT-059, respectively. At 1 month post-TBI biospecimens were collected from 5 naïve animals, 4 animals administered FB, 5 animals each from 10.5 Gy BBT-059 and 11.5 Gy BBT-059 groups, and all three remaining animals from the 12.5 Gy BBT-059 group. At 6 months post-TBI, biospecimens were collected from 5 naïve animals, 4 animals administered FB prior to TBI, 5 animals each from the 10.5 Gy and 11.5 Gy BBT-059 groups, and 5 animals from the 12.0 Gy BBT-059 group. At 12 months post-TBI, biospecimens were collected from all remaining animals (4 naïve, 4 FB, 4 10.5 Gy BBT-059, 3 11.5 Gy BBT-059, and 1 12.0 Gy BBT-059). The single remaining animal from the 12.0 Gy BBT-059 group at the 12 month time point was excluded as no statistical analysis could be conducted.

### Peripheral blood cell counts up to 12 months post-TBI

During the DRF study (days1–30 post-exposure) neither body weights nor blood was collected; however, in the long term study, blood (20uL via the submandibular vein) was collected at 1.5, 5, 7, 8, 9, 11, and 12 months and body weights were collected at the same time points (Supplemental Figs. 1 and 2). Over the course of the DEARE study, no significant changes were observed in body weights. Blood was analyzed for white blood cells (WBC), neutrophils (NEU), lymphocytes (LYM), platelets (PLT), and red blood cells (RBC). All counts are summarized in Supplemental Tables S1-5 and in Supplemental Figs. S1–2. Results are described in Supplemental *i*nformation. In FB-treated survivors, lower WBC counts were observed at 1.5 months (0.993 ± 1.072 vs. 3.343 ± 0.896, p = 0.0223) and 5 months (3.960 ± 1.682 vs. 9.014 ± 0.967, p = 0.0123) compared to naïve animals. In addition, LYM counts were lower in FB-treated animals compared to naïve animals at 1.5 months (0.030 ± 0.000 vs. 2.453 ± 0.786, p < 0.0001) and 5 months (0.723 ± 0.724 vs. 6.470 ± 1.970, p = 0.0004). LYM counts were also lower in the group administered BBT-059 prior 12 Gy irradiation compared to naïve at 1.5 months (0.067 ± 0.042 vs. 2.453 ± 0.786, p < 0.0001). Finally, PLT counts were lower in FB-treated animals compared to naïve animals at 1.5 months (125.67 ± 50.06 vs. 820.75 ± 209.10, p = 0.0033) and they were lower in animals administered BBT-059 prior to 12 Gy irradiation compared to naïve at the same time point (334.00 ± 226.90 vs. 820.75 ± 209.10, p = 0.0399).

### Hematopoietic progenitor cells clonogenic assay

**Figure 2:**
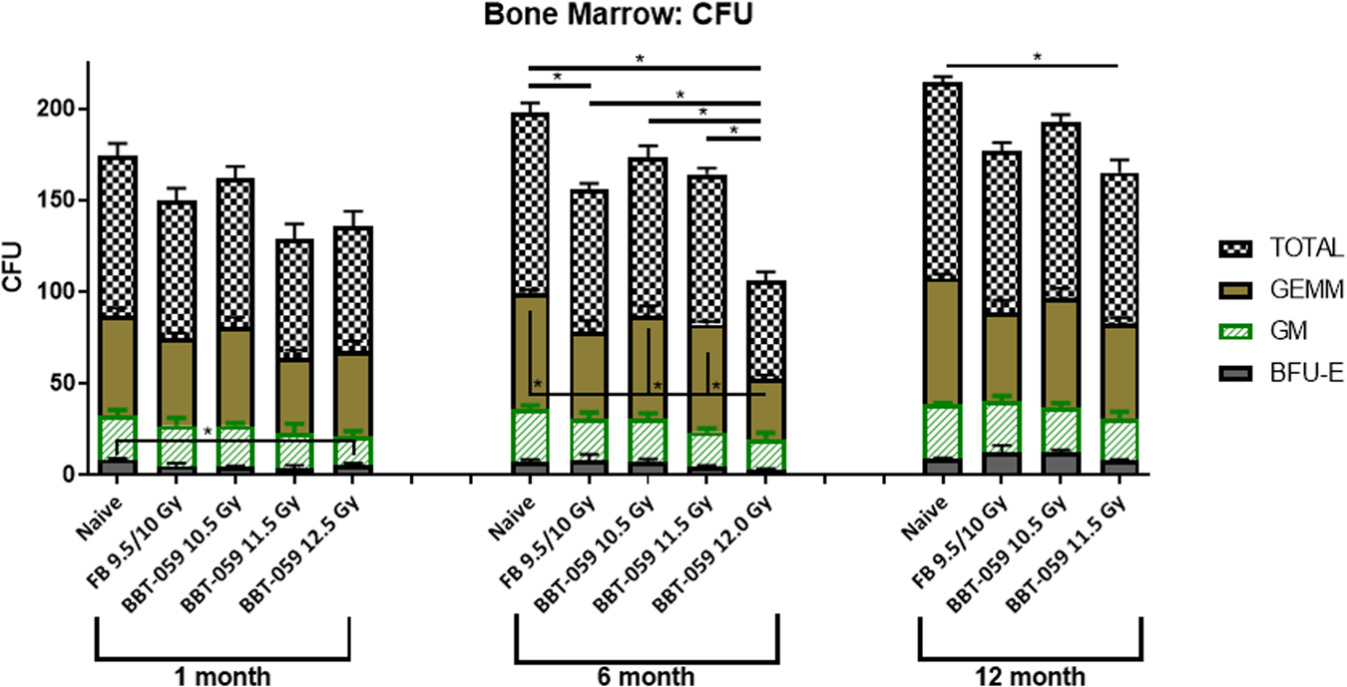
Hematopoietic progenitor cell recovery in CD2F1 mice up to 12 months post-lethal dose of TBI. CFU assay was done to analyze the clonogenic potential of bone marrow cells. Colony forming units (CFU) were assayed on 1, 6 and 12 months after TBI. Cells from the femurs of three different animals were pooled, counted, and each sample plated in duplicate to be scored 14 days after plating. Data are expressed as mean ± Standard error of mean (SEM).

At 1, 6, and 12 months post-TBI, femurs were collected from animals, the bone marrow isolated, and a colony forming unit (CFU) assay performed. At 1 month post-TBI, no statistical differences for granulocyte, erythrocyte, monocyte, megakaryocyte progenitor (GEMM), and granulocyte-macrophage progenitor (GM), or total counts were observed (Fig. 2). Statistically lower counts of burst-forming unit-erythroid (BFU-E) were observed after 1 month in the 11.5 Gy BBT-059 group compared to the naïve control (3.750 ± 2.986 vs. 8.500 ± 1.000, p = 0.0470). At 6 months post-TBI, no statistical differences in the counts for BFU-E or GM were observed. Counts for GEMM were statistically lower in the 12.0 Gy BBT-059 group compared to naïve (33.500 ± 3.317 vs 62.750 ± 4.193, p = 0.0009), the 10.5 Gy BBT-059 group (33.500 ± 3.317 vs 56.000 ± 10.801, p = 0.0088), and the 11.5 Gy BBT-059 group (33.500 ± 3.317 vs 58.750 ± 3.304, p < 0.0001). Several differences were observed at 6 months for the total counts as well, the 12.0 Gy BBT-059 group had statistically lower counts than all other groups (compared to: naïve 53.000 ± 9.309 vs 99.000 ± 10.231, p < 0.0001, FB group 53.000 ± 9.309 vs 77.750 ± 7.274, p = 0.0147, 10.5 Gy BBT-059 group 53.000 ± 9.309 vs 86.750 ± 12.176 p = 0.0011, and 11.5 Gy BBT-059 group 53.000 ± 9.309 vs 82.000 ± 6.928 p = 0.0043). Also at 6 months, the total counts in the FB group was statistically lower than the naïve group (77.750 ± 7.274 vs 99.000 ± 10.231, p = 0.0404). At 12 months post-TBI, no statistical differences were observed among any groups for GEMM, BFU-E, or GM counts, however the total counts in the 11.5 Gy BBT-059 group were statistically lower than the naïve group (82.500 ± 13.964 vs 107.250 ± 5.965, p = 0.0185).

### Monitoring hepatic and renal panel in serum long term after radiation

**Figure 3:**
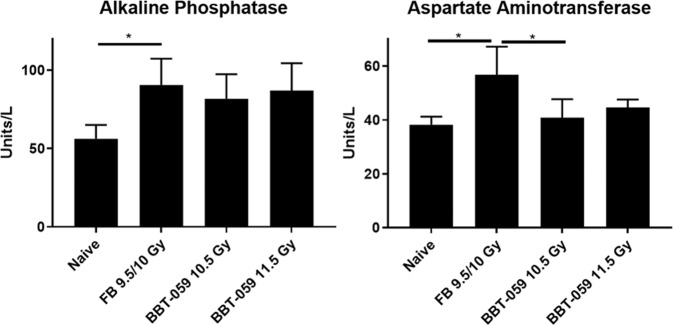
Serum biochemistry: Alkaline phosphatase (ALKP) and aspartate aminotransferase (AST) of irradiated mice administered FB or BBT-059 24 h prior to TBI at 10.0, 10.5, 11.5, 12 and 12.5 Gy and naïve (0 Gy) collected at 12 months post-TBI.

At the 12 month time point, a terminal blood collection was performed, serum separated, and biochemical analysis performed. In this analysis, the concentrations of several renal and hepatic analytes were determined (data not shown). Among those analytes, alkaline phosphatase (ALKP) and aspartate aminotransferase (AST) were found to be significantly upregulated in the FB group (Fig. 3). The concentration of ALKP in the serum samples of the naïve animals was 56.25 ± 8.85 U/L vs 90.50 ± 16.86 U/L (p = 0.0308) for the FB group. By contrast, the ALKP concentration in the serum samples of the 10.5 Gy and 11.0 Gy BBT-059 groups was 81.00 ± 15.61 U/L and 87.00 ± 17.44 U/L, respectively. These values were not statistically different from the naïve control group, the FB group, or one another. AST serum levels in the naïve animals was determined to be 38.25 ± 2.99 U/L which was statistically lower than the AST serum levels in the FB group (56.75 ± 10.40 U/L, p = 0.0109). In addition, AST serum levels were also statistically higher in the group administered FB compared to the 10.5 Gy BBT-059 group (40.80 ± 6.87 U/L, p = 0.0201). By contrast, the AST levels in the 11.5 Gy BBT-059 group (44.67 ± 2.89 U/L) were not statistically different from any group. No significant changes were observed among the groups for the other analytes investigated (creatinine, alanine aminotransferase, blood urea nitrogen, total protein, and phosphorus) (Fig. 3).

### Histopathological analysis of major organs

#### Sternum

**Figure 4:**
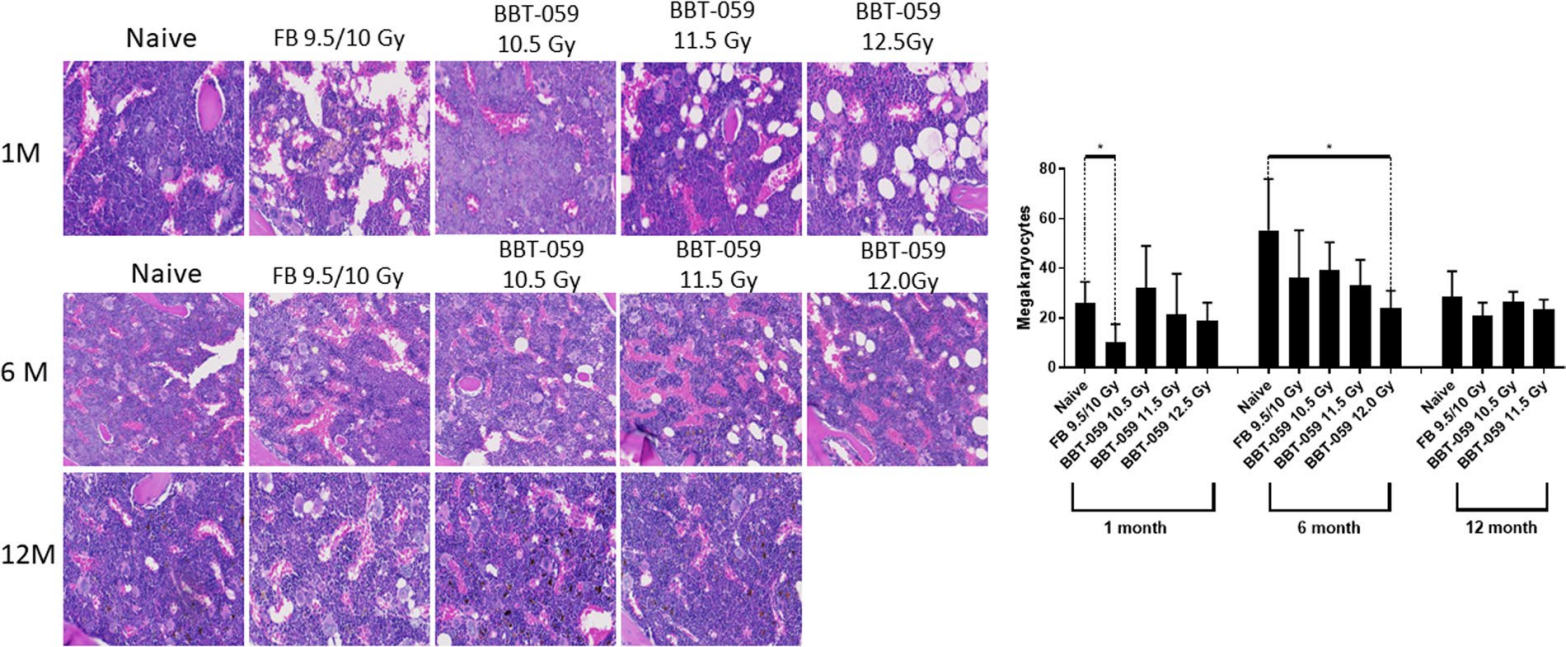
BBT-059 administration promoted sternal bone marrow hematopoietic cell recovery after lethal dose of TBI. Increases in bone marrow cellularity and megakaryocytes were observed after 30 days in the Drug treatment group. There was no difference between FB and BBT-059 treated groups at 6 and 12 months post-TBI. Data represented are mean ± standard error of the mean (SEM).

Sternal sections from the FB group stained with hematoxylin & eosin (H&E) showed decreased cellularity compared to both the naïve groups and all groups administered BBT-059 (Fig. 4). Among the BBT-059 groups, the 10.5 Gy BBT-059 group showed the highest cellularity, and cellularity appeared to decrease as the radiation dose got higher. In addition, megakaryocytes were counted for these sections, and there was a statistically significant decrease in the number of megakaryocytes for the FB group compared to naïve at 1 month (10.000 ± 7.528 vs 26.250 ± 8.221, p = 0.027) and for the 12.0 Gy BBT-059 group compared to naïve at 6 months (23.750 ± 7.228 vs 55.250 ± 20.646, p = 0.028). Similar to the total bone marrow cellularity, there was a trend towards decreased megakaryocyte numbers as the radiation dose was increased amongst the groups administered BBT-059.

#### Lung

**Figure 5:**
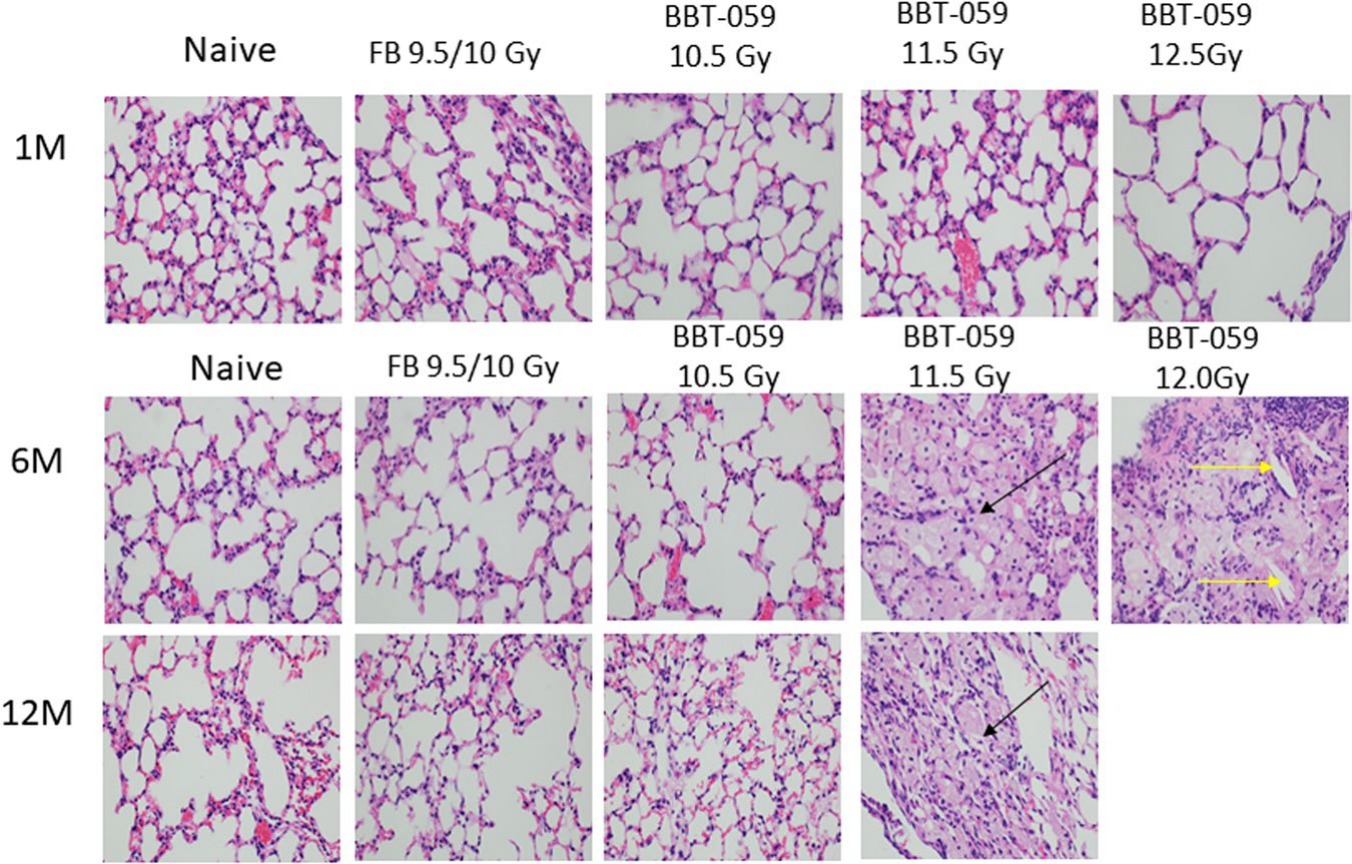
Lung histology demonstrates cholesterol clefts and Histiocytes. Differences between the histiocytic pneumonitis and the normal lungs. These photos of lungs taken at 40× magnification at the objective (total 400×) Photos BBT-059 11.5–6 M, BBT-059 11.5–12 M; and BBT-059 12–6 M; all show histiocytic pneumonitis. The large “foamy” cells identified with arrows are all histiocytes. The yellow arrow identifies a “cholesterol cleft”. They commonly occur in areas of the lungs with these foamy macrophages, often in association with a lot of proteinaceous material. Please note, it is possible that the histiocytic pneumonitis is simply a background lesion that would have occurred in these animals regardless of exposure.

Lungs were collected from animals in each of the groups at 1, 6, and 12 months, excluding the 12.0 Gy BBT-059 group at 12 months as all these animals had perished prior to this time point. These lungs were sectioned and stained with H&E (Fig. 5), periodic acid-schiff (PAS), and Masson’s Trichrome before inspection by a certified pathologist. The major findings in the lungs was several instances of histiocytosis (black arrows) in three animals out of four in each group at 11.5 Gy and 12 Gy BBT-059 after 6 months, however after 12 months only one animal out of three had histiocytosis at 11.5 Gy BBT-059 group. Cholesterol clefts (yellow arrows) were present in a single animal at 6 months from the 12.0 Gy BBT-059 group. In order to assess the extent of the lesions, the following grading scale was used: a minimal rating indicates 1–10% of the lung area was affected, mild had 11–24% affected, moderate had 25–45% affected, and severe had >45% affected. No lesions were identified in any of the lungs collected at 1 month post-TBI. By 6 months post-TBI, several lesions were noted, sections from the lungs of 4 animals were noted as minimal including 3 out of 4 of the animals in the 12.0 Gy BBT-059 group and 1 animal from the 11.5 Gy BBT-059 group. Sections from one animal were scored as mild, 1 out of 4 animals in the 11.5 Gy BBT-059 group. Finally, sections from one animal in the 11.5 Gy BBT-059 group was scored as moderate. All together 3 out of 4 animals in the 11.5 Gy and 12.0 Gy BBT-059 groups showed lesions. By 12 months, the only lesion noted was in 1 out of the 3 animals from the 11.5 Gy BBT-059 group and it was scored as minimal. In addition to these findings, sections from a single mouse (out of 5) at the 12 month time point from the 10.5 Gy BBT-059 group indicated pulmonary carcinoma. The instance of pulmonary carcinoma is not uncommon for mice of advanced age but the disease progression could also be related to the radiation exposure.

#### Heart

Hearts were collected from animals in each of the groups at 1, 6, and 12 months, excluding the 12.0 Gy BBT-059 group as all these did not survive to this time point. At the 6 month time point, sections from several irradiated mice (but not the naïve group) indicated a small amount of pericardial mineralization. This was concluded to be a background lesion often termed dystrophic cardiac calcinosis. A neutrophilic infiltration was observed in single mouse from the 10.5 Gy BBT-059 group and was thought to be a precursor to the aforementioned dystrophic cardiac calcinosis. No other histological changes were observed.

#### Liver

There were several findings detected in the liver, but all were clinically insignificant and most likely were background lesions. There was no indication of drug toxicity or irradiation effects at any time point. Most of the mice had some glycogen in their hepatocytes, which is a common background lesion in mice euthanized in the morning and indicates they were eating during the night. At higher radiation doses some mice had infiltrates of mononuclear cells in the liver, but these infiltrates are common background lesions in older mice. No other major histological changes were observed.

### Irradiation increases senescence in mouse kidney

**Figure 6:**
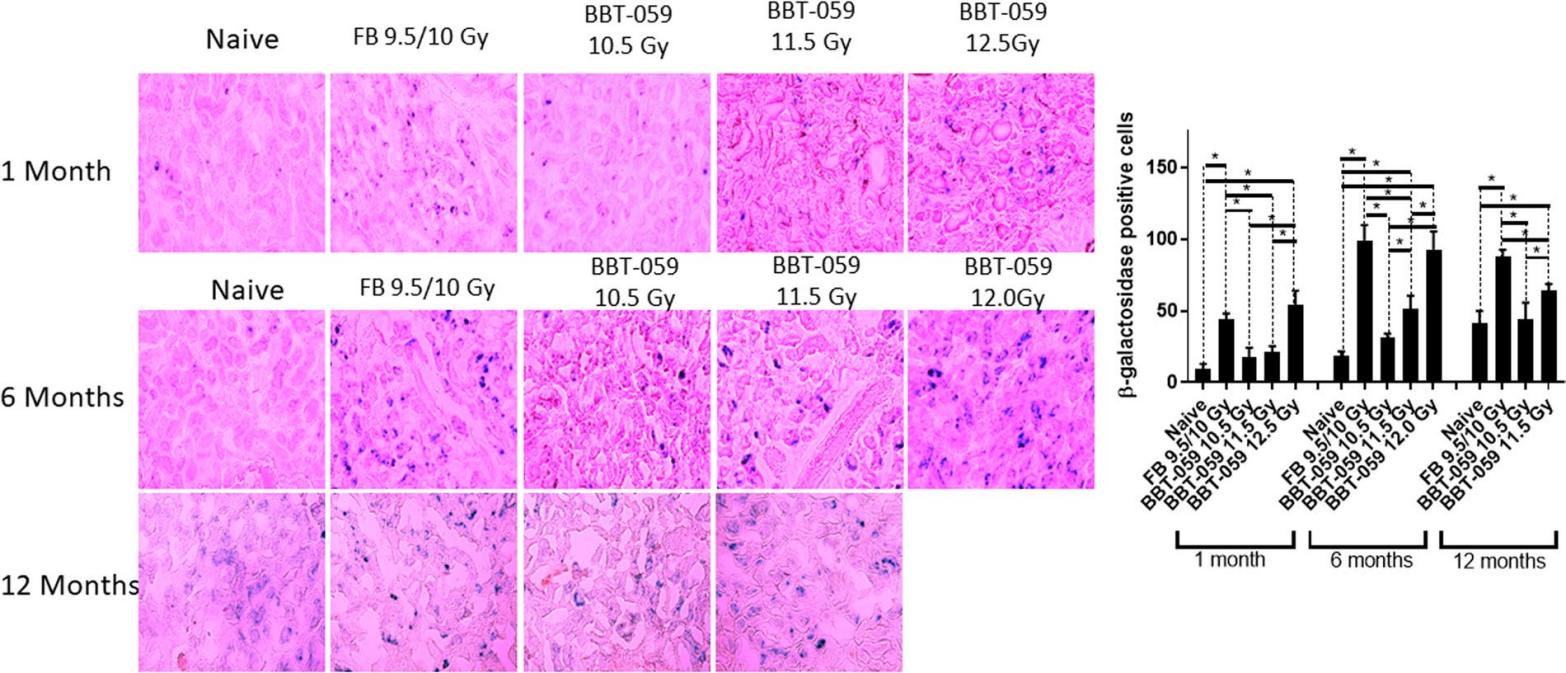
Radiation-induced cellular senescence in kidney. Increased numbers of senescence-associated β-galactosidase (SA β-gal)-positive cells in the kidneys administered FB or BBT-059 24 h prior to irradiation at the highest radiation dose (12.5 Gy). Kidneys isolated from mice at 1, 6 and 12 months were stained for SA β-gal. Cells stained blue are positive for SA β-gal. Significant differences are indicated (*p < 0.05). Scale bars = 20 µm.

The expression of SA β-gal, a senescence marker, was analyzed in sections from kidney (Fig. 6) collected from animals in all groups at 1, 6, and 12 months post-TBI with the exception of the 12 month, 12 Gy BBT-059 group as all animals had perished.

In kidney at 1 month post-TBI, there was an increase in the number of β-gal positive cells in the kidneys of animals in the FB and 12.5 Gy BBT-059 groups. For the FB group, the number of β-gal positive cells was statistically higher than the number in the naïve group (43.750 ± 4.349 vs 9.250 ± 3.594, p < 0.0001), the 10.5 Gy (43.750 ± 4.349 vs 17.750 ± 6.397, p = 0.0003), and 11.5 Gy (43.750 ± 4.349 vs 21.000 ± 4.320, p = 0.0010) BBT-059 groups. In addition, the number of β-gal positive cells in the 12.5 Gy BBT-059 group was statistically higher than the naïve (53.750 ± 10.210 vs 9.250 ± 3.594, p < 0.0001), the 10.5 Gy (53.750 ± 10.210 vs 17.750 ± 6.397, p < 0.0001), and 11.5 Gy (53.750 ± 10.210 vs 21.000 ± 4.320, p < 0.0001) BBT-059 groups.

At the 6 month time point, there were several statistically significant findings. The number of β-gal positive cells in the naïve group was lower than the FB group (18.750 ± 2.872 vs. 99.250 ± 10.436, p < 0.0001), the 11.5 Gy (18.750 ± 2.872 vs. 51.500 ± 8.963, p = 0.0005), and the 12.0 Gy (18.750 ± 2.872 vs. 92.750 ± 12.553, p < 0.0001) BBT-059 groups. In addition to having higher β-gal positive cells than the naïve group, the number of β-gal positive cells in the FB group was also higher than the number in the 10.5 Gy (99.250 ± 10.436 vs. 31.500 ± 2.380, p < 0.0001) or 11.5 Gy (99.250 ± 10.436 vs. 51.500 ± 8.936, p < 0.0001) BBT-059 groups. The 10.5 Gy BBT-059 group had fewer β-gal positive cells than the 11.5 Gy (31.500 ± 2.380 vs 51.500 ± 8.936, p = 0.0317) or 12.0 Gy (31.500 ± 2.380 vs 92.750 ± 12.553, p < 0.0001) BBT-059 groups. Finally, the 11.5 Gy BBT-059 group had fewer β-gal positive cells than the 12.0 Gy BBT-059 group (51.500 ± 8.963 vs 92.750 ± 12.553, p = 0.0017).

At the 12 month time point, significant findings were noted. The number of β-gal positive cells was lower in the naïve group than in the FB group (41.500 ± 8.386 vs 87.500 ± 5.066, p < 0.0001) and the 11.5 Gy BBT-059 group (41.500 ± 8.386 vs 64.250 ± 4.349, p = 0.0073). The number of β-gal positive cells in the FB group was higher than the number in the 10.5 Gy (87.500 ± 5.066 vs 44.000 ± 11.633, p < 0.0001) or the 11.5 Gy, 87.500 ± 5.066 vs 64.250 ± 4.349, p = 0.0063) BBT-059 groups. Finally the 11.5 Gy BBT-059 group had more β-gal positive cells than the 10.5 Gy BBT-059 group (64.250 ± 4.349 vs 44.000 ± 11.633, p = 0.0172).

In addition to these findings in the kidneys, we also collected liver and brain and conducted a similar analysis (Supplemental Figs. S3 and S4) however minimal senescence was observed in these organs.

### Loss of E cadherin following radiation

**Figure 7:**
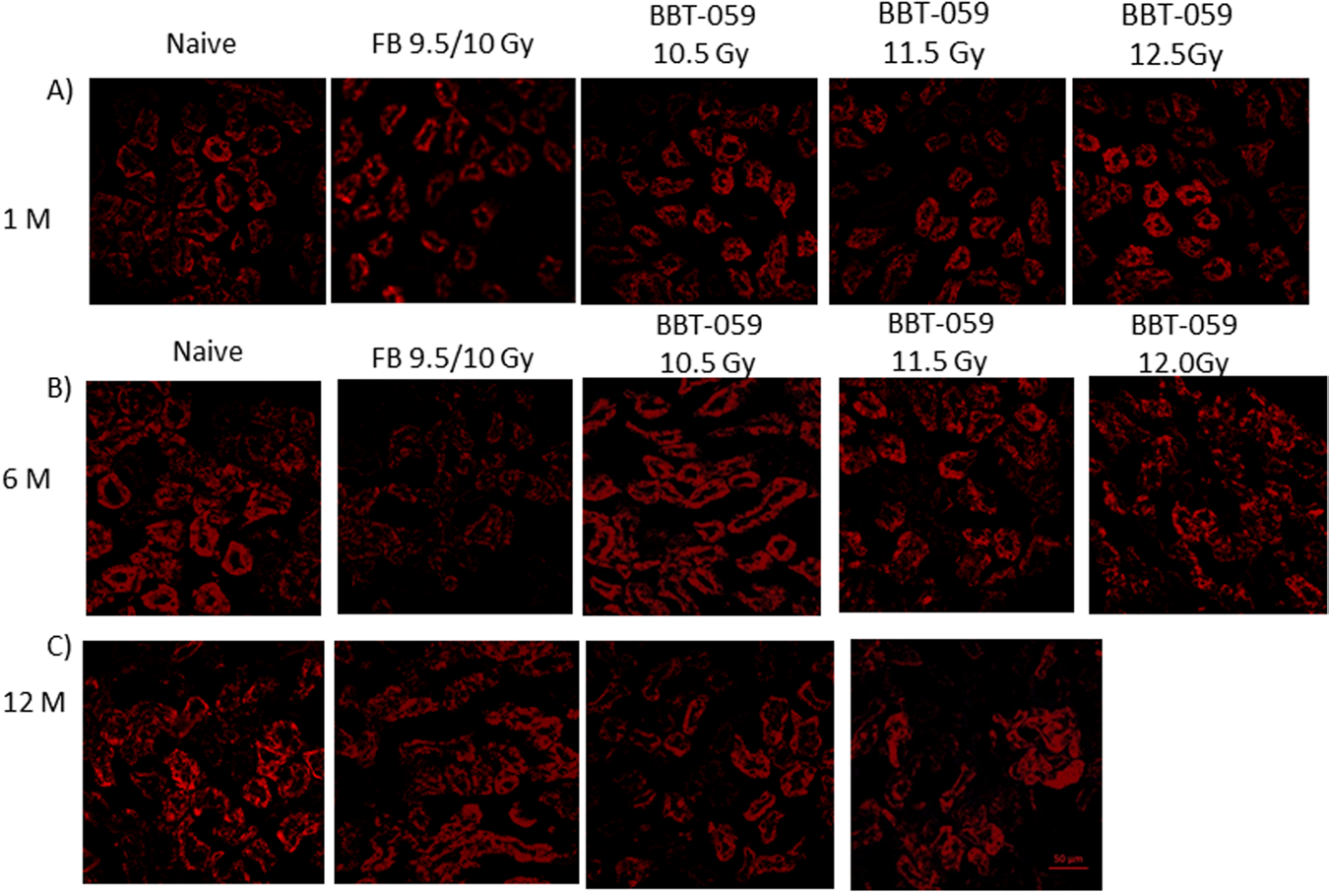
E cadherin is decreased in kidney post-TBI. Immunofluorescent staining showing a decrease in E cadherin in the group administered FB 24 h prior to TBI at 6 months compared to the naïve and BBT-059 treated groups.

E-cadherin is a calcium-dependent cell-cell adhesion molecule which is important in the formation of adherens junctions to bind cells with each other and have an essential role in tissue formation, epithelial cell behavior, and suppression of cancer. On the surface of all epithelial cells, E-cadherin molecules are linked to the actin cytoskeleton through interactions with catenins in the cytoplasm. To determine the E-cadherin expression, immunohistochemistry (IHC) was performed on kidney sections collected at 1, 6 and 12 months post-TBI. Representative images (Fig. 7) showed that E-cadherin (red) expression is reduced in the kidneys from animals from the FB group at six months. However in the BBT-059 groups, there was increased expression of E-cadherin in all groups. E-cadherin is involved in fibrosis which is an active extracellular matrix (ECM) biosynthetic process and represents the terminal pathway of chronic failure of many organs.

### Western blot analysis

**Figure 8:**
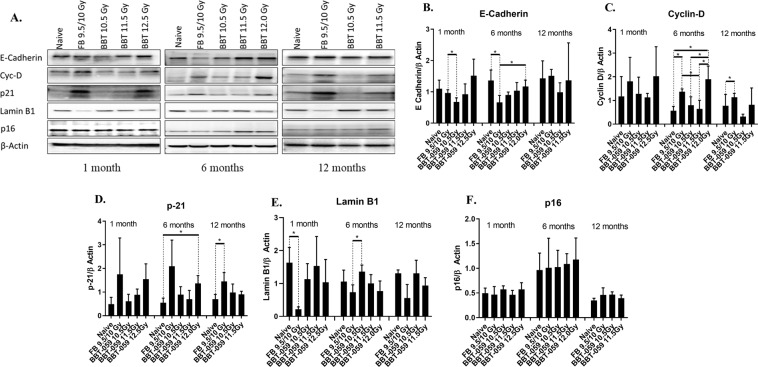
Western blot analysis: (**A**) Expression of E-cadherin, Cyc D, P21, Lamin B1 and p16 expression spleens collected at 1, 6 and 12 month from all groups **B**–**F**) Quantification from three independent western blots and the bar graph shows the quantification of the ratio of protein of interest and its respective β-Actin control.

Spleens were collected from animals at 1, 6, and 12 months post-TBI, lysates made, and western blot analysis performed. At the 1 month time point, Lamin B1 levels were decreased in the FB group compared to the naïve (0.219 ± 0.069 vs 1.630 ± 0.463, p = 0.0064). Also at the one month time point, E-cadherin levels were lower in the 10.5 Gy BBT-059 group compared to the FB group (0.795 ± 0.534 vs 1.799 ± 0.461, p = 0.0387) (Fig. 8).

At the 6 month time point, several statistically significant differences were observed, Lamin B1 levels were decreased in the FB group compared to the 10.5 Gy group (0.733 ± 0.226 vs 1.359 ± 0.200, p = 0.0229). E-cadherin levels were statistically lower in the FB group compared to naïve (0.662 ± 0.222 vs. 1.360 ± 0.337, p = 0.0400) and the 12.0 Gy BBT-059 group (0.662 ± 0.222 vs. 1.172 ± 0.202, p = 0.0420). Cyclin–D levels were significantly elevated in the FB group compared to naïve (1.371 ± 0.118 vs. 0.569 ± 0.178, p = 0.0029) and the 11.5 Gy BBT-059 group (1.371 ± 0.118 vs. 0.645 ± 0.363, p = 0.0302). Cyclin-D levels were also significantly elevated in the 12.0 Gy BBT-059 group compared to naïve (1.897 ± 0.559 vs. 0.569 ± 0.178, p = 0.0173), the 10.5 Gy (1.897 ± 0.559 vs. 0.802 ± 0.355, p = 0.0458) and 11.5 Gy (1.897 ± 0.559 vs. 0.645 ± 0.363, p = 0.0314) BBT-059 groups. Increased p21 expression has been observed in senescent cells^29^ and p21 levels were statistically elevated in the 12.0 Gy BBT-059 group compared to naïve (1.364 ± 0.336 vs. 0.552 ± 0.194, p = 0.0222). The levels of p21 also appeared to be elevated in the FB group compared to naïve; however, it was not significantly different (2.091 ± 1.108 vs. 0.552 ± 0.194, p = 0.0768) (Fig. 8). The levels of P16 in the 11.5 and 12 Gy BBT-059 groups appeared elevated compared to other groups but was not significant.

At the 12 month time point, only three statistically significant differences were observed, Lamin B1 levels were significantly less in FB group as compared to naïve (0.559 ± 0.409 vs. 1.306 ± 0.106, p = 0.0376), Cyclin-D levels were higher in the FB group compared to the 10.5 Gy BBT-059 group (1.137 ± 0.164 vs 0.317 ± 0.103, p = 0.0019) and p21 levels were elevated in the FB group compared to naïve (1.451 ± 0.374 vs. 0.700 ± 0.201, p = 0.0375) (Fig. 8).

## Discussion

Here we describe the use of a murine model to determine the DRF for the promising radiation countermeasure BBT-059 and investigate DEARE following administration of BBT-059 or its control. As we showed previously and confirmed in this work, BBT-059 greatly enhances survival in male CD2F1 mice^9^. The current results indicate a dose reduction factor of 1.28 with a 95% confidence interval of 1.14–1.57, with 50% mortality of male CD2F1 occurring at 11.81 Gy compared to 9.25 Gy for animals administered formulation buffer. Based on the previously determined safety profile and the relatively high DRF value^30^, BBT-059 is an extremely promising countermeasure in rodent. Aside from providing exceptional increases in survival even at supralethal doses of radiation one of the main objectives of the current study was to study long-term effects in animals that have been administered the drug as a prophylactic countermeasure. In the context of protecting military and first responders who might become occupationally exposed to radiation in efforts related to a radiological event. It is import to consider both the short term survival of these individuals and potential long-term health effects that might arise.

Our study shows no appreciable weight change over 12 months post-TBI period in the surviving animals compared to healthy age matched controls (Supplementary Fig. S1). Visually it was noted that the fur of animals that were exposed to radiation became grey at a quicker rate than the naïve controls, consistent with previous reports of hypopigmentation following exposure to gamma-rays^31^.

Biochemical analysis of serum at 12 months post-TBI revealed elevated levels of ALKP and AST in the FB group whereas no significant changes were observed in the animals administered BBT-059, although no significant ALKP levels did appear to be elevated in the 2 groups administered BBT-059 compared to the naïve control group. In previous studies, elevated ALKP levels have been noted in mice shortly after irradiation (24–72 h) in liver tissue^32^ and up to 30 days in the serum^33^. Previously increased levels of ALKP were observed in the serum of patients characterized with proliferating bone marrow^34^. The current results indicate a homeostatic change in the levels of these analytes following irradiation that is present as far out to 12 months which seems to be ameliorated by administration of BBT-059 prior to TBI. A direct comparison to previous studies is difficult, at the time of blood collection for serum analysis the animals were ~65 weeks (12 months post-TBI) whereas such analysis in previous studies reported on animals ranging in age from 45–130 days^35–37^.

CFU analysis of the femoral bone marrow demonstrated a loss of CFUs in the 12.0 Gy BBT-059 group. This analysis was based on the data at 6 months, as these animals (12 Gy BBT-059) did not survive to 12 months. By 12 months the total number of all CFU cell types was statistically lower in the 11.5 Gy BBT-059 group as well, but there was no statistical difference in any of the individual counts (BFU, GEMM, or GM) at this or any other time point. We do not know the exact cause of mortality in the 12.0 Gy BBT-059 group but it is possible that the inability to produce sufficient viable bone marrow progenitor cells contributed to their mortality. The evaluation of cellularity and megakaryocyte numbers in the sternal sections from each of the groups indicated increased recovery with BBT-059 treatment compared to FB.

Histopathology of sections of the lungs in this study revealed the highest incidence of lesions at the 6 month post-TBI time point. At this time point a total of 6 out of 8 animals administered BBT-059 24 h prior to TBI (3 out of 4 irradiated at 11.5 Gy and 3 out of 4 irradiated at 12.0 Gy) had noticeable lesions deemed to be histiocytic pneumonitis. These findings were further clarified by Gomori Methenamine-Silver GMS staining not to be an infection of *Pneumocystis murina*. The lesions can be common in older animals but the finding that the lesions were not present in the age matched healthy animals or in animals irradiated at a lower dose indicates this finding could be connected to the radiation dose received. Within the study, there was a single instance of pulmonary carcinoma which is a common lesion in older mice but might also result from the 12 months after radiation exposure as spontaneous lesion. Finally, a single instance of cholesterol clefts (yellow arrows, Fig. 5) was present in one out of 4 of the animals in the 12.0 Gy BBT-059 group. This lesion can be indicative of an inflammatory response in the lung and could be related to radiation exposure or it could also be a background lesion as it was only observed in a single animal in the study. Histopathology of the sections from the heart and liver did not reveal any lesions that were thought to be study related based on the analysis by a certified pathologist.

Radiation causes oxidative stress and damages the DNA, thereby causing apoptosis or premature senescence in the tissues and cells. Many short term studies have shown that ionizing radiation causes early senescence; 12 month long term *in vivo* studies are lacking. In this study SA β-gal staining was examined in kidney, liver, and brain. SA β-gal expression is generally used to recognize senescent cells due to its robust correlation with senescence and relatively simple detection. We showed that there were more β –gal positive cells in kidneys of animals administered FB or BBT-059 prior to higher doses of radiation (11.5 and 12.0 Gy) compared to naive; however, at lower radiation doses BBT-059 appears to protect the cells from senescence, as indicated by fewer SA β –gal positive cells compared to animals administered FB. The specific physiological association of SA β -gal with senescence is not clear^38,39^. Minimal senescence was observed in the brain or liver from any time point amongst all the groups.

To further investigate senescence, we conducted western blot analyses on lysates prepared from spleens from the various groups with lamin-B1 and anti-p21 antibody.

Cancer biologist and many researchers ascribe the relationship between abnormal expression of lamin and cancer subtype by examining alterations in lamin expression in different types of cancers. Loss of lamin B1 plays a key role in lung cancer and it has been demonstrated that lamin B1 levels were decreased in patients of lung cancer^40^. Epithelial–mesenchymal transition, tumor growth, cell migration and metastasis is promoted by lamin B1 silencing in lung epithelial cells^40^. It was shown that lamin B1 is decreased in murine and primary human cells when they are induced to senescence by replicative exhaustion, DNA damage, or oncogene expression^24^. Western blot results from our study showed decreased expression of lamin B1 in FB treated animals compared to naïve at 1,6 and 12 months. In recent years, many studies reported that lamin B1 expression is reduced in senescence, which postponements cell proliferation and endorses cellular senescence via an Rb-dependent p53 mechanism^41,42^. Lamin B1 loss was described as biomarker of senescence both in culture and *in vivo*^24^.

We also investigated another recognized marker of cellular senescence, p21^43^. P53 transcriptionally controls p21 and acts as a cyclin-dependent kinase inhibitor that promotes cell cycle arrest and senescence^44^. Western blot analysis of p21 protein expression showed a trend toward increased levels in the group administered FB and also in the 12.0 Gy BBT-059 treated group. Elevated expression of p21 has been previously reported following exposure to ionizing radiation^45^. An earlier report demonstrated increased expression of senescence-related markers in liver, brain, and lung tissues several weeks after exposure to ionizing radiation^20^. In contrast, the current study implies that the senescence markers examined reached increased expression levels after 30 days and remained relatively unchanged through the end of the 12 month study. Continuous rise in the expression of p21 level is reported to be related with senescence induced by ionizing radiation in hematopoietic stem cells and human dental pulp stem cells^46,47^. Therefore, the persistent rise in the expression of p21 and β-gal in the FB group and in the BBT-059 groups at higher radiation doses observed in the current long term study may be strong evidence that exposure to ionizing radiation can cause organ senescence or aging in mice. Among the groups administered BBT-059, the ones that received lower doses of TBI showed lower senescence.

Authors have shown that adhesion proteins like E-cadherin are actively involved in mesenchymal to epithelial (MET) and epithelial to mesenchymal (EMT) transitions, which play an important role in cancer progression and tissue fibrosis^48^. The current immunohistochemistry results from kidney sections showed that E- cadherin expression appeared lower in the FB group and higher in the naïve and BBT-059 administered groups at six months. Results were validated by doing western blots of spleen lysates, which exhibited the similar results for E-cadherin expression. E-cadherin is a cell adhesion molecule which plays an important role in maintaining renal epithelial integrity and polarity. Many studies have shown that E-cadherin is significantly downregulated in acute kidney injury, however its function in acute kidney injury is unknown. The pathological role played by E- cadherin in kidney damage is not well-understood and further work is needed to elucidate its involvement. Decreased E-cadherin levels leading to the loss of cell-cell adhesion could lead to fibrosis.

In addition, decreased expression of E-cadherin has been demonstrated in the kidneys of patients with glomerulonephritis as well as diabetic and chronic allograft nephropathies^49–51^. Morphogenetic tumor effects may be related to disruption in E-cadherin levels. Decreases in E-cadherin expression or the loss of its ability to localize in cellular compartments is often detected during tumor development^52–54^. Another oncogene involved in the pathogenesis of cancer is Cyc D and in our study cyclin-D expression was significantly higher in spleens of the FB and 12.0 Gy BBT-059 groups at 6 months compared to naïve and the groups administered BBT-059 at lower radiation doses.

In this study, we demonstrated the excellent enhancement in 30 day survival following administration of BBT-059 to CD2F1 male mice with a DRF of 1.28. Mice from this experiment were monitored for long term (up to 12 months post-TBI) to study DEARE and we found that these mice did not have significant changes in body weight over this period. There was accelerated recovery of blood cell counts, hematopoietic progenitor colony forming units, and sternal megakaryocytes in animals treated with BBT-059. Histological changes to the lungs including histiocytic pneumonitis were observed in animals irradiated at higher doses regardless of treatment (FB or BBT-059). Increased senescence was observed in animals administered either vehicle or drug prior to TBI. Decreased E-cadherin levels were observed in animals at six months administered vehicle 24 h prior to TBI, and modulation of these changes in drug-treated survivors. Taken together, this study reports that animals surviving long term from lethal and supralethal doses of radiation did not show any changes in the major organs. In addition, we show that BBT-059 might be regulating the expression of E-cadherin, Cyc D, p21 and lamin B1 post-radiation exposure. However, further studies are necessary to explore the mechanism of drug in radiation protection.

## Materials and Methods

### Animals and irradiation

The animal study was conducted as described previously^9,55,56^. The study was carried out in accordance with the recommendations in the Guide for the care and use of laboratory animals of the National Institutes of Health. Specific approval was obtained from the institutional animal care and use committee (IACUC) at the Armed Forces Radiobiology Research Institute (AFRRI). 8–10 weeks old CD2F1 male mice were purchased from Envigo, USA. The mice were housed in the AFRRI vivarium, which is accredited by the Association for Assessment and Accreditation of Laboratory Animal Care-International. Animals are provided acidified water (pH ~2.5) and feed (Harlan Teklad Global Rodent Diet 8604) *ad libitum*. Experimental animals were identified by tail tattoo and housed 4 per box in sterile cages. Animals were acclimatized for 1–2 weeks before the start of the study. The animal rooms were maintained at 21 ± 2 °C and 50 ± 10% relative humidity with 10–15 cycles of fresh air hourly and a 12:12 h light:dark cycle.

Mice were irradiated bilaterally (simultaneously) at an estimated dose rate of 0.6 Gy/min in the Cobalt-60 gamma-irradiation facility at the Armed Forces Radiobiology Research Institute, Bethesda, MD^9^. An alanine / Electron Spin Resonance (ESR) dosimetry system (American Society for Testing and Material Standard E 1607) has been used to quantity the dose rates in the cores of acrylic phantoms (1 inch in diameter and 3 inches long) located in all vacant slots of the exposure rack in the Lucite restraint boxes. ESR signal was determined by calibration curve based on standard calibration dosimeters provided NIST, Gaithersburg, MD. The calibration curve was confirmed by inter-comparison with the National Physical Laboratory (NPL) in the United Kingdom. The corrections applied to the measured dose rates in phantoms were for decay of the Co-60 source and for a small difference in mass-energy absorption coefficients for water and soft tissue at the Co-60 energy level. The radiation field was previously reported to be uniform within ±2%^57,58^.

### Dose reduction factor and DEARE studies

To determine the dose reduction factor animals were placed into one of twelve groups. Groups 1–6 were administered the FB (10 mM sodium phosphate, 1% sucrose, 4% mannitol, pH 6.2) for BBT-059 (0.1 mL, subcutaneous injection) and groups 7–12 were administered BBT-059 (0.3 mg/kg in formulation buffer, 0.1 mL, subcutaneous injection) 24 h prior to TBI. Each group was then irradiated at a different radiation doses as follows: 7.5, 8.0, 8.5, 9.0, 9.5, 10.0 Gy for groups 1–6, respectively and 10.5, 11.0, 11.5, 11.75, 12.0, and 12.5 Gy for groups 7–12, respectively. Animals were monitored up to 3 times daily and animals that were found in moribund condition were euthanized based on guidelines established with the AFRRI IACUC and documented. Additionally, animals that were found dead were removed from the cages and documented. At 30 days post-TBI the proportion of remaining animals at each radiation dose was used to perform a probit analysis (IBM SPSS Statistics 25.0) of the groups administered FB or BBT-059 and the LD50/30 value for each was determined. Surviving animals from the DRF studies were kept and monitored for up to 12 months post-TBI to study DEARE. In addition a group of non-irradiated, age-matched, naïve animals were kept and monitored for up to 12 months. Biospecimens were collected from these animals as outlined in the sections that follow.

### Blood and tissue collection

At various time points in the study (1.5, 5, 7, 9, 11, and 12 months) blood (~20uL) was collected from animals via the submandibular vein. This sample was used to collect complete blood counts (CBC) using a Heska Element HT5 Veterinary Hematology Analyzer (Heska/Cuattro Loveland, CO). At 1 month, 6 months, and 12 months after radiation exposure, blood was terminally collected under anesthesia (5% Isoflurane, Baxter International Inc., Deerfield, IL) for serum collection in BD (Beckton, Dickinson and Company, Franklin Lakes, NJ) microtainer tubes, left to clot 1 hour and separation via centrifugation 2400xg for 10 min). Animals were euthanized following blood collection via cervical dislocation and femurs, sterna, spleens, hearts, lungs, and kidneys collected. These tissues were either collected in 10% buffered formalin, 4% paraformaldehyde, or flash frozen depending on the analysis to be performed.

### Hematopoietic progenitor clonogenic assay

Clonogenic assay of mouse bone marrow cells was quantified in semisolid cultures using 1 mL of Methocult GF + system for mouse cells (Stem Cell Technologies Inc., Vancouver, BC) according to the manufacturer’s instructions and as described previously^9,56^. Colony forming units (CFU) were quantified in samples collected at 1, 6 and 12 months. Bone marrow cells were pooled from the femurs from three different animals, washed with Iscove Modified Dulbecco Media two times and seeded at 1 to 5 ×10^4^ cells per dish on 35-cm cell culture dishes (BD Biosciences). All samples were plated in duplicate and scored after 14 days. Granulocyte-erythrocyte-monocyte-macrophage CFU (CFU-GEMM), burst-forming unit-erythroid (BFU-E) and colony-forming unit granulocyte macrophage (CFU-GM) were identified and quantified following the manufacturer’s instructions. Colonies were counted after 14 days using a Nikon TS100F microscope. Data are expressed as mean ± standard error of mean.

### Histopathology

Liver, kidney, heart and sternum were collected for histology at 1, 6, and 12 months. Tissues were fixed in 10% buffered formalin solution for at least 24 h. Sterna were decalcified in 12–18% sodium EDTA (pH 7.4–7.5) for 3 h. Before embedding into paraffin all tissues were dehydrated using graded ethanol (70% to 100% ethanol). Hematoxylin and eosin (H&E) was used to stain the sections and images were captured with an Olympus BX41 camera and analyzed with Adobe Photoshop. A blinded hstopathological evaluation for all the samples were conducted by a board-certified veterinary pathologist.

### Senescence associated β-galactosidase (SA β-gal) staining

SA β-gal staining of kidneys, livers, and brains was performed per kit instructions (Cell Signaling Senescence β-Galactosidase Staining Kit #9860). Briefly, tissue samples were snap-frozen in an optimal cutting temperature compound Tissue-Tek® OCT™ Compound (Sakura Finetek Europe B.V., The Netherlands). Fresh frozen sections (10-µm thick) were cut with a cryostat (Leica Biosystems, Germany), and mounted on slides. The slides were fixed for 15 minutes in 1x fixative solution, followed by PBS washes, and incubated overnight with beta-galactosidase staining solution in a dry incubator at 37 °C with no CO_2_ supply to prevent false positivity. Sections were viewed under a microscope for blue color development and were photographed with an Olympus BX41 camera^59^. After photographing the slides, positive cells for SA β-gal were counted using ImageJ software (ver. 1.38; National Institutes of Health, USA).

### Immunohistochemistry

Immunohistochemistry was performed as described previously^55^. For tissue sections, samples were fixed with 4% paraformaldehyde (PFA) at room temperature, then washed with 1× PBS. Samples were incubated overnight in 20% sucrose at 4 °C, and then in 30% sucrose for 4 hours at room temperature. Samples were blocked with a 1% BSA solution for 30 minutes and then embedded in Tissue-Tek® OCT™ Compound (Sakura Finetek Europe B.V., The Netherlands) and 12 μm sections prepared. Samples were incubated with primary antibodies (E-cadherin from Cell Signaling #3195 S) at a concentration of 1:100 overnight at 4 °C and with secondary antibodies (1:500) for two hours at room temperature. Confocal images were obtained using a Zeiss 700 camera.

### Western blot

Western blotting was performed as previously described^55^. At 1 month, 6 months, and 12 months spleens were isolated, snap-frozen and were stored at −80 °C. Spleens were homogenized and lysed in ice cold RIPA buffer (Sc24948A Santa Cruz Biotechnology) in the presence of protease inhibitors (Sigma P8340). The homogenates were centrifuged at 12,000 × g for 10 min at 4 °C, and the total protein concentrations were determined using a BCA protein assay (Thermo Fisher). After normalizing the concentrations, 4X Laemmli sample buffer (BIORAD #1610747) was added to achieve a 1X final concentration. Samples were denatured by boiling at 100 °C for 5 minutes and were run on 4–12% SDS-PAGE gels at 80 volts for 60 minutes, then transferred to PVDF membranes by semi-dry transfer (Trans-Blot® Turbo™ Transfer System, BIORAD). Membranes were blocked with 4% bovine serum albumin (BSA) in 1X TBST and incubated with rabbit monoclonal anti-E-cadherin (Cell Signaling, 3195 S), rabbit monoclonal anti-Cyclin D1 (Cell Signalling, 2978 S), and rabbit polyclonal antibody anti-P21 (Thermofisher, 14671581) at 4 °C overnight with gentle agitation. Membranes were then washed 3 times with TBST and incubated with horseradish peroxidase - conjugated secondary antibody for 60 minutes. Membranes were washed 3 times with TBST and then incubated with enhanced chemiluminescence reagents (GE Healthcare RPN2106) and finally developed using (ChemiDoc™ Imaging Systems, BIORAD). Beta-actin (Santa Cruz Sc47778) was used as a loading control. The intensities of specific bands corresponding to the proteins of interest were measured by using Image-J software.

### Statistical analysis

Normal-quantile (Q-Q) plots were constructed to analyze the normality of the data. For multiple groups a parametric one-way analysis of variance (ANOVA) followed by Fisher’s least significant difference (LSD) post-hoc test was applied. For comparison of two different groups, an unpaired student-t test was applied, and for non-normal data, Kruskal-Wallis H test (non-parametric) followed by Mann-Whitney-U tests were applied.

## Supplementary information

Supplementary information.
