# Supplementary material for: Delayed effects of acute whole body lethal radiation exposure in mice pre-treated with BBT-059
Source: Sci Rep. 2020 Apr 22;10:6825. doi: 10.1038/s41598-020-63818-7 (PMC7176697; doi:10.1038/s41598-020-63818-7)
Supplement: Supplementary file 1 — Supplementary information. [file 41598_2020_63818_MOESM1_ESM.docx]

**Delayed effects of acute whole body lethal radiation exposure in mice pre-treated with BBT-059**

**Neel K. Sharma^1*#^, Gregory P. Holmes-Hampton^1*^, Vidya P. Kumar^1^, Shukla Biswas^1^, Kefale Wuddie^1^, Sasha Stone^1^, Zemenu Aschenake^1^, William L. Wilkins^1^, Christine M. Fam^2^, George N. Cox^2^, Sanchita P. Ghosh^1#^**

Supplemental material:

WBC (Supplemental Table S1): At 1.5 months post-TBI the only significantly different values for WBC counts were between the naïve group and the group administered formulation buffer 24h prior to TBI (p=0.0223). At 5 months post-TBI several statistically different values were observed including: WBC counts for the naïve group where higher compared to the group administered formulation buffer 24h prior to TBI (p=0.0123), counts for the naïve group were lower compared to the group administered BBT-059 24h prior to TBI at 11.5 Gy (p=0.0213), the counts for the group administered formulation buffer 24h prior to TBI were lower compared to the group administered BBT-059 24h prior to TBI at 10.5 Gy (p=0.0011), and the group administered BBT-059 24h prior to TBI at 11.5 Gy (p<0.0001), and the group administered BBT-059 24h prior to TBI at 11.5 Gy were higher than the group administered BBT-059 24h prior to TBI at 12 Gy.

At 7 months post-TBI, the WBC counts for the group administered BBT-059 24h prior to TBI at 11.5 Gy were statistically higher than all other treatment groups: counts for the group administered BBT-059 24h prior to TBI at 11.5 Gy vs. naïve (p=0.0008), counts for the group administered BBT-059 24h prior to TBI at 11.5 Gy vs. the counts for the group administered formulation buffer 24h prior to TBI (p=0.0003), counts for the group administered BBT-059 24h prior to TBI at 11.5 Gy vs. counts for the group administered BBT-059 24h prior to TBI at 10.5 Gy (p=0.0006), and counts for the group administered BBT-059 24h prior to TBI at 11.5 Gy vs. counts for the group administered BBT-059 24h prior to TBI at 12.0 Gy (p=0.0040). At 8 months only one significant difference was observed, the WBC counts for the naïve group was lower than the counts for the group administered BBT-059 24h prior to TBI at 11.5 Gy (p=0.0238).

At 9 months post-TBI, WBC counts for the group administered BBT-059 24h prior to TBI at 10.5 Gy were statistically higher than the following groups: naïve (p=0.0132), the group administered formulation buffer 24h prior to TBI (p=0.0050), and the group administered BBT-059 24h prior to TBI at 11.5 Gy (p=0.0413). No other statistical differences were observed. At both 11 and 12 months post TBI no statistical differences for WBC counts were observed among any of the groups.

NEU (Supplemental Table S2): At 1.5 months post-TBI the only significantly different values for NEU counts were between the naïve group and the group administered BBT-059 24h prior to TBI at 12.0 Gy (p=0.0436). At 5 months post-TBI the only significantly different values for NEU counts were between the naïve group and the group administered BBT-059 24h prior to TBI at 11.5 Gy (p=0.0439). At 7 months post-TBI there were two statistically different values for NEU: the counts of the naïve group was lower than the counts of the group administered BBT-059 24h prior to TBI at 11.5 Gy (p=0.0370) and the counts of the group administered BBT-059 24h prior to TBI at 10.5 Gy were lower than the counts of the group administered BBT-059 24h prior to TBI at 11.5 Gy (p=0.0441). At 8 months post-TBI no statistical differences were observed for NEU counts among any of the groups. At 9 months post-TBI, NEU counts for the naïve group were statistically lower than the counts for the groups administered formulation buffer 24h prior to TBI (p=0.0248) and for the group administered BBT-059 24h prior to TBI at 10.5 Gy (p=0.0025). In addition, statistically higher levels were observed in the group administered BBT-059 24h prior to TBI at 10.5 Gy compared to the group administered BBT-059 24h prior to TBI at 11.5 Gy (p=0.0156). At both 11 and 12 months post TBI no statistical differences were observed for NEU counts among any of the groups.

LYM (Supplemental Table S3): At 1.5 months post-TBI several statistically significant differences were observed. LYM in the naïve group were higher than all other groups: counts for the naïve group compared to the counts for the group administered formulation buffer 24h prior to TBI (p<0.0001), the counts for the naïve group compared to the counts for the group administered BBT-059 24h prior to TBI at 10.5 Gy (p=0.0002), the counts for the naïve group compared to the counts for the group administered BBT-059 24h prior to TBI at 11.5 Gy (p=0.0001), and the counts for the naïve group compared to the counts for the group administered BBT-059 24h prior to TBI at 12.0 Gy (p<0.0001). No other statistical differences for LYM counts among the groups were observed.

At 5 months post-TBI the LYM counts for the naïve group were statistically higher than the group administered formulation buffer 24h prior to TBI (p=0.0004). In addition the LYM counts for the group administered formulation buffer 24h prior to TBI were statistically lower than the LYM counts for all groups that were administered BBT-059 24h prior to TBI: compared to the group administered BBT-059 24h prior to TBI at 10.5 Gy (p=0.0001), compared to the group administered BBT-059 24h prior to TBI at 11.5 Gy (P<0.0001), and compared to the group administered BBT-059 24h prior to TBI at 12.0 Gy (p=0.0140). The LYM counts for the group administered BBT-059 24h prior to TBI at 11.5 Gy weres statistically higher than the counts for the group administered BBT-059 24h prior to TBI at 12.0 Gy (p=0.0151).

At 7 months post-TBI the LYM counts were statistically higher for the group administered BBT-059 24h prior to TBI at 11.5 Gy compared to all other groups: compared to the naïve group (p=0.0050), compared to the group administered formulation buffer 24h prior to TBI (p=0.0005), compared to the group administered BBT-059 24h prior to TBI at 10.5 Gy (p=0.0031), and compared to the group administered BBT-059 24h prior to TBI at 12.0 Gy (p=0.0034). No other statistical differences for LYM counts among the groups were observed.

At 8 months post-TBI the LYM counts were statistically higher for the group administered BBT-059 24h prior to TBI at 11.5 Gy compared to all other groups: compared to the naïve group (p<0.0001), compared to the group administered formulation buffer 24h prior to TBI (p<0.0001), compared to the group administered BBT-059 24h prior to TBI at 10.5 Gy (p=0.0007), and compared to the group administered BBT-059 24h prior to TBI at 12.0 Gy (p=0.0001). In addition, the LYM counts for the group administered BBT-059 24h prior to TBI at 10.5 Gy were statistically higher than both the group administered formulation buffer 24h prior to TBI (p=0.0462) and the group administered BBT-059 24h prior to TBI at 12.0 Gy (p=0.0001).

At 9 months post-TBI, the only statistical difference for LYM counts was the observation of higher counts in the group administered BBT-059 24h prior to TBI at 10.5 Gy compared to the group administered formulation buffer 24h prior to TBI (p=0.0258). No other statistical differences for LYM counts among the groups were observed. At both 11 and 12 months post TBI no statistical differences were observed for LYM counts among any of the groups.

PLT (Supplemental Table S4): At 1.5 months post-TBI the PLT counts for the group administered formulation buffer were statistically lower than the naïve group (p=0.0033). In addition the PLT counts for the group administered BBT-059 24h prior to TBI at 12.0 Gy were lower than the naïve group (P=0.0399). At 5 months post-TBI no statistical differences for PLT counts among the groups were observed. At 7 months post-TBI PLT counts were statically lower in the group administered formulation buffer compared to the naïve group (p=0.0454), the group administered BBT-059 24h prior to TBI at 11.5 Gy (p=0.0074), and the group administered BBT-059 24h prior to TBI at 12.0 Gy (p=0.0238). At 8, 9, 11, and 12 months post-TBI no statistical differences in PLT counts were observed among any of the groups.

RBC (Supplemental Table S5): At none of the time points was there a significant difference in the RBC counts among any of the groups.

| **Table S1: WBC counts** | | | | | | | | |
| --- | --- | --- | --- | --- | --- | --- | --- | --- |
| **Radiation Dose (Gy)** | Treatments | 1.5 Months | 5 Months | 7 Months | 8 Months | 9 Months | 11 Months | 12 Months |
| **0** | Naïve | 3.343 ± 0.896 | 9.014 ± 0.967 | 7.985 ± 0.553 | 7.385 ± 0.572 | 6.100 ± 3.100 | 7.993 ± 3.140 | 10.150 ± 0.999 |
| **9.5 & 10.0** | Formulation Buffer | 0.993 ± 1.072 | 3.960 ± 1.682 | 7.540 ± 2.142 | 9.960 ± 8.204 | 5.350 ± 2.008 | 11.750 ± 5.598 | 9.183 ± 5.212 |
| **10.5** | BBT-059 | 2.120 ± 0.400 | 10.670 ± 1.517 | 8.122 ± 1.201 | 11.126 ± 0.883 | 11.408 ± 1.684 | 12.354 ± 0.853 | 11.432 ± 2.217 |
| **11.5** | BBT-059 | 2.503 ± 0.505 | 13.147 ± 3.848 | 12.980 ± 1.773 | 17.816 ± 0.909 | 6.573 ± 0.842 | 10.347 ± 1.537 | 8.180 ± 3.395 |
| **12.0** | BBT-059 | 3.010 ± 1.330 | 7.757 ± 3.740 | 8.397 ± 0.519 | 9.397 ± 1.238 | 6.700 ± 0.170 | N/A | N/A |

**Supplemental Table S1:** WBC counts at various time points for the different groups of animals, counts are reported as mean ± SD, all units are 10^3^ cells/ml. For all groups and time points, n=3-10. When insufficient animals survived N/A was reported.

| **Table S2: NEU counts** | | | | | | | | |
| --- | --- | --- | --- | --- | --- | --- | --- | --- |
| **Radiation Dose (Gy)** | Treatments | 1.5 Months | 5 Months | 7 Months | 8 Months | 9 Months | 11 Months | 12 Months |
| **0** | Naïve | 0.810 ± 0.146 | 1.583 ± 0.341 | 1.995 ± 0.206 | 1.640 ± 0.336 | 1.485 ± 0.500 | 1.925 ± 0.845 | 2.313 ± 0.249 |
| **9.5 & 10.0** | Formulation Buffer | 0.813 ± 0.911 | 1.775 ± 0.601 | 2.460 ± 0.435 | 3.333 ± 3.877 | 3.120 ± 0.378 | 3.530 ± 1.718 | 2.967 ± 1.967 |
| **10.5** | BBT-059 | 1.243 ± 0.376 | 2.020 ± 0.720 | 2.070 ± 0.398 | 2.986 ± 0.178 | 3.598 ± 1.039 | 2.938 ± 0.238 | 3.050 ± 0.325 |
| **11.5** | BBT-059 | 1.583 ± 0.531 | 2.649 ± 0.628 | 2.892 ± 0.530 | 3.562 ± 0.052 | 1.787 ± 0.140 | 3.703 ± 0.846 | 2.850 ± 1.315 |
| **12.0** | BBT-059 | 1.483 ± 1.224 | 1.998 ± 1.111 | 2.683 ± 0.392 | 3.170 ± 0.779 | 2.960 ± 0.028 | N/A | N/A |

**Supplemental Table S2:** NEU counts at various time points for the different groups of animals, counts are reported as mean ± SD, all units are 10^3^ cells/ml. For all groups and time points, n=3-10. When insufficient animals survived N/A was reported.

| **Table S3: LYM counts** | | | | | | | | |
| --- | --- | --- | --- | --- | --- | --- | --- | --- |
| **Radiation Dose (Gy)** | Treatments | 1.5 Months | 5 Months | 7 Months | 8 Months | 9 Months | 11 Months | 12 Months |
| **0** | Naïve | 2.453 ± .786 | 6.470 ± 1.970 | 5.663 ± 0.359 | 5.500 ± 0.386 | 4.378 ± 2.577 | 5.845 ± 2.249 | 7.440 ± 0.900 |
| **9.5 & 10.0** | Formulation Buffer | 0.030 ± 0.000 | 0.723 ± 0.724 | 4.573 ± 2.615 | 4.415 ± 3.205 | 1.610 ± 2.693 | 6.993 ± 2.673 | 5.350 ± 3.910 |
| **10.5** | BBT-059 | 0.718 ± 0.095 | 7.376 ±1.890 | 5.682 ± 0.957 | 7.624 ± 0.668 | 7.320 ± 2.306 | 8.904 ± 0.617 | 7.866 ± 2.048 |
| **11.5** | BBT-059 | 0.513 ± 0.371 | 8.774 ± 3.064 | 9.594 ± 1.117 | 13.048 ± 0.784 | 4.510 ± 0.934 | 6.320 ± 0.440 | 4.937 ± 2.050 |
| **12.0** | BBT-059 | 0.067 ± 0.042 | 4.990 ± 3.057 | 5.117 ± 0.767 | 5.630 ± 0.265 | 3.085 ± 0.106 | N/A | N/A |

**Supplemental Table S3:** LYM counts at various time points for the different groups of animals, counts are reported as mean ± SD, all units are 10^3^ cells/ml. For all groups and time points, n=3-10. When insufficient animals survived N/A was reported.

| **Table S4: PLT counts** | | | | | | | | |
| --- | --- | --- | --- | --- | --- | --- | --- | --- |
| **Radiation Dose (Gy)** | Treatments | 1.5 Months | 5 Months | 7 Months | 8 Months | 9 Months | 11 Months | 12 Months |
| **0** | Naïve | 820.75 ± 209.10 | 1093.40 ± 318.20 | 1141.50 ± 145.63 | 1271.50 ± 58.92 | 994.25 ± 320.06 | 1208.00 ± 100.64 | 1255.25 ± 182.78 |
| **9.5 & 10.0** | Formulation Buffer | 125.67 ± 50.06 | 718.86 ± 471.34 | 727.25 ± 113.69 | 978.00 ± 226.98 | 686.67 ± 540.7 | 1091.33 ± 314.49 | 1057.67 ± 311.08 |
| **10.5** | BBT-059 | 480.25 ± 285.39 | 761.63 ± 201.61 | 1035.20 ± 239.64 | 1215.80 ± 59.87 | 1196.60 ± 28.34 | 1283.60 ± 52.65 | 1229.20 ± 110.24 |
| **11.5** | BBT-059 | 575.25 ± 53.47 | 910.10 ± 277.84 | 1235.60 ± 207.45 | 1106.40 ± 112.45 | 1071.00 ± 236.18 | 1247.00 ± 25.63 | 1051.50 ± 392.63 |
| **12.0** | BBT-059 | 334.00 ± 226.90 | 708.60 ± 331.28 | 1222.33 ± 177.16 | 1133.67 ± 246.61 | 1322.00 ± 18.39 | N/A | N/A |

**Supplemental Table S4:** PLT counts at various time points for the different groups of animals, counts are reported as mean ± SD, all units are 10^3^ cells/ml. For all groups and time points, n=3-10. When insufficient animals survived N/A was reported.

| **Table S5: RBC counts** | | | | | | | | |
| --- | --- | --- | --- | --- | --- | --- | --- | --- |
| **Radiation Dose (Gy)** | Treatments | 1.5 Months | 5 Months | 7 Months | 8 Months | 9 Months | 11 Months | 12 Months |
| **0** | Naïve | 9.030 ± 0.436 | 9.735 ± 2.900 | 10.230 ± 0.435 | 9.210 ± 0.897 | 9.315 ± 1.188 | 8.128 ± 1.723 | 9.103 ± 1.487 |
| **9.5 & 10.0** | Formulation Buffer | 6.835 ± 1.880 | 10.443 ± 1.080 | 10.310 ± 0.222 | 9.668 ± 0.100 | 9.883 ± 1.927 | 9.048 ± 1.216 | 9.125 ± 0.578 |
| **10.5** | BBT-059 | 9.005 ± 0.576 | 8.819 ± 2.039 | 9.878 ± 0.374 | 10.062 ± 0.241 | 9.612 ± 0.216 | 9.654 ± 0.755 | 9.544 ± 0.539 |
| **11.5** | BBT-059 | 7.803 ± 0.853 | 10.034 ± 2.091 | 9.914 ± 0.301 | 9.762 ± 0.263 | 7.860 ± 1.464 | 9.143 ± 0.898 | 7.783 ± 3.654 |
| **12.0** | BBT-059 | 7.767 ± 0.759 | 8.829 ± 2.779 | 10.273 ± 0.115 | 10.237 ± 0.668 | 9.275 ± 0.233 | N/A | N/A |

**Supplemental Table S5:** RBC counts at various time points for the different groups of animals, counts are reported as mean ± SD, all units are 10^6^ cells/ml. For all groups and time points, n=3-10. When insufficient animals survived N/A was reported.

**Supplemental Figure S1**

**Supplemental Figure S1: Body weights of the animals throughout the long term study**. Body weights were not measured in the initial 30 days post-TBI. n=3-5 depending on available animal numbers.

**Supplemental Figure S2**

**Supplemental Figure S2:** **Peripheral blood cells recovery:** White blood cells (WBC), neutrophils (NEU), lymphocytes (LYM), platelets (PLT), and red blood cells (RBCs) of irradiated mice administered FB or BBT-059 24h prior to TBI at 10.0, 10.5, 11.5, 12 and 12.5 Gy and naïve (0 Gy). Data represented are mean ± standard error of the mean (SEM).

**Supplemental Figure S3**

**
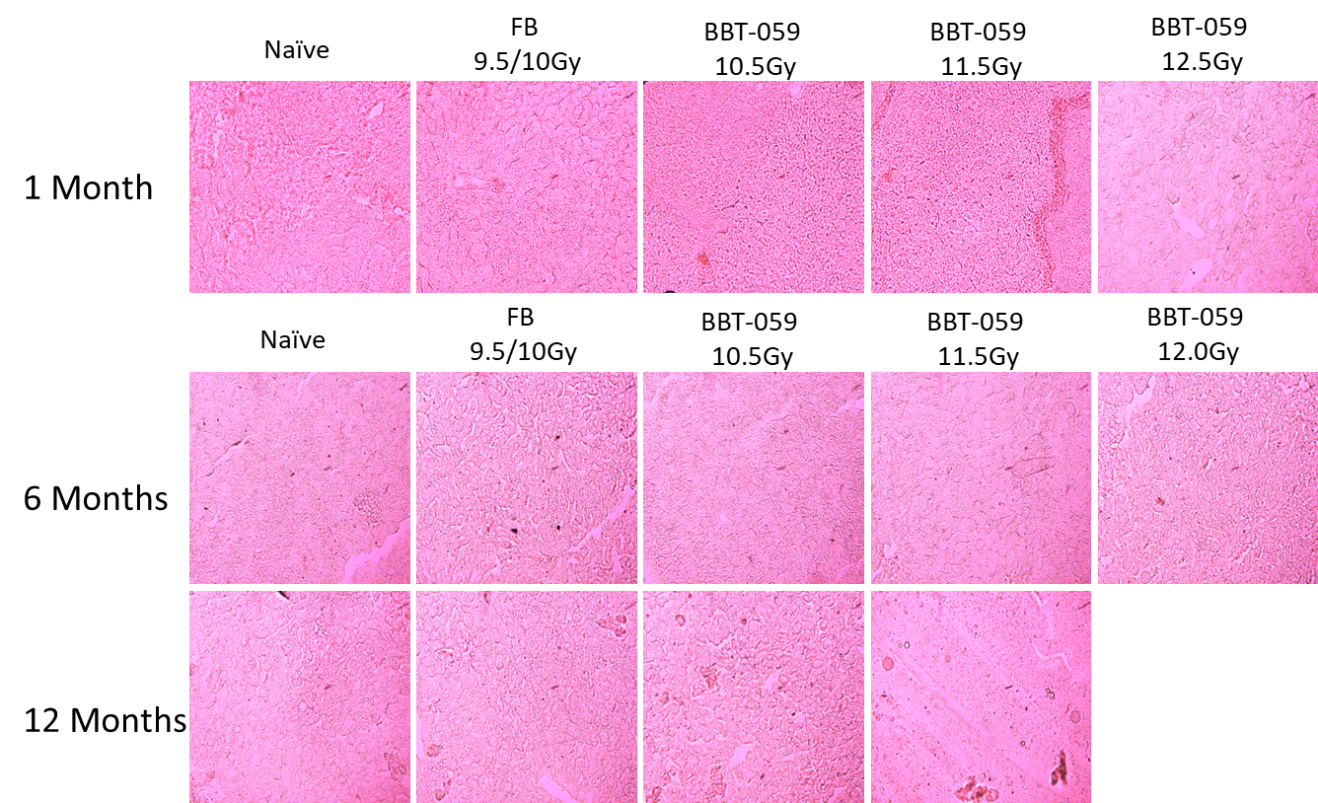
**

**Supplemental Figure S3:** **Radiation-induced cellular senescence in liver from CD2F1 male mice.** Minimal changes were observed among the different collection time points, radiation doses and treatment.

**Supplemental Figure S4**

**
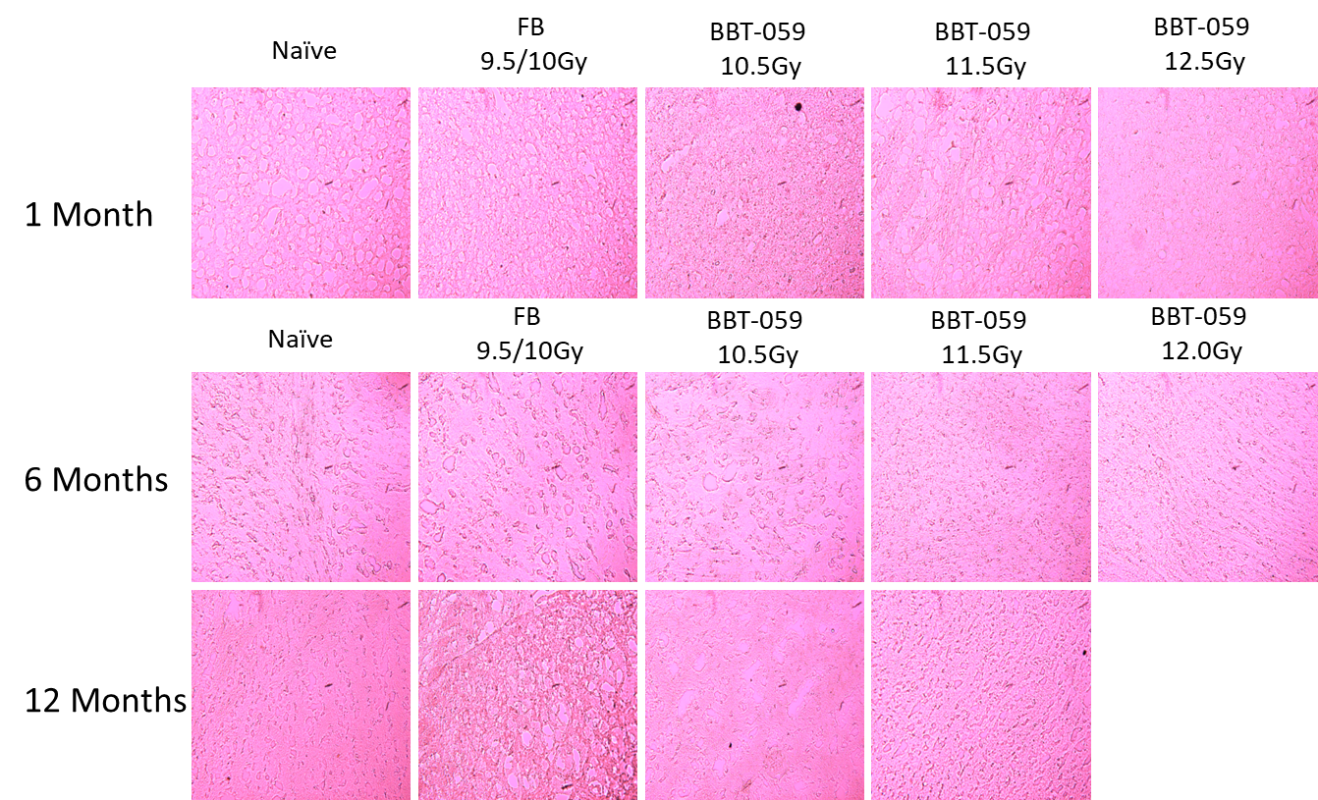
**

**Supplemental Figure S4:** **Radiation-induced cellular senescence in brain from CD2F1 male mice.** Minimal changes were observed among the different collection time points, radiation doses and treatment.
